# Mitigating the Spread and Translocation of *Salmonella* Enteritidis in Experimentally Infected Broilers under the Influence of Different Flooring Housing Systems and Feed Particle Sizes

**DOI:** 10.3390/microorganisms9040874

**Published:** 2021-04-18

**Authors:** Marwa F. E. Ahmed, Amr Abd El-Wahab, Jan-Philip Kriewitz, Julia Hankel, Bussarakam Chuppava, Christine Ratert, Venja Taube, Christian Visscher, Josef Kamphues

**Affiliations:** 1 Hygiene and Zoonoses Department, Faculty of Veterinary Medicine, Mansoura University, Mansoura 35516, Egypt; Marwa.Ahmed@tiho-hannover.de; 2 Nutrition and Nutritional Deficiency Diseases Department, Faculty of Veterinary Medicine, Mansoura University, Mansoura 35516, Egypt; Amr.Abd.El-Wahab@tiho-hannover.de; 3 Institute for Animal Nutrition, University of Veterinary Medicine Hannover, Foundation, Bischofsholer Damm 15, D-30173 Hannover, Germany; sekretariat-tierernaehrung@tiho-hannover.de (J.-P.K.); julia.hankel@tiho-hannover.de (J.H.); bussarakam.chuppava@tiho-hannover.de (B.C.); service-tierernaehrung@tiho-hannover.de (C.R.); josef.kamphues@tiho-hannover.de (J.K.); 4 BEST 3 Geflügelernährung GmbH, D-27239 Twistringen, Germany; v.taube@best-3.de

**Keywords:** *Salmonella*, broiler, floor designs, diets physical form

## Abstract

This study aimed to evaluate the influences of different flooring designs and feed particle sizes on the spread of *Salmonella* (*S.*) in broiler chickens. Birds (*n* = 480) were allocated to four different housing systems (fully littered with and without floor heating, partially and fully slatted flooring with sand bath) and two dietary treatments (finely and coarsely ground diets) in 24 boxes. Two broilers per box were experimentally infected with *S.* Enteritidis (8.00 log_10_ CFU/bird) at d 17. *Salmonella* prevalence in caecal contents and the liver was highest in broilers housed on fully slatted floor until d 36/37 (88.1% and 91.5%, respectively), and lowest in litter flooring (caecal content 64.4%) and litter flooring with floor heating (liver 61.7%). In turn, broilers on littered flooring expressed the lowest *Salmonella* counts in caecal content at d 36/37 (2.21 ± 1.75 log_10_ CFU/g), partial slatted flooring the highest (3.76 ± 1.46 log_10_ CFU/g). The mean *Salmonella* count in the caecal content was significantly lower for birds fed a coarsely ground diet (0.96 and 1.94 log_10_ CFU/g) than a finely ground diet (5.07 and 3.34 log_10_ CFU/g) at d 23 and d 36/37, respectively (*p* < 0.0001). Slatted flooring with a sand bath did not show advantages in terms of *Salmonella* reduction, whereas the coarsely ground diet markedly reduced the spread of *Salmonella*.

## 1. Introduction

The consumption of poultry meat has been increasing worldwide for years [1,2]. One risk linked to the production of poultry meat are infections with zoonotic agents such as *Salmonella*. Salmonellosis is the second most common cause of zoonotic diseases in humans within the European Union and is often linked to the consumption of poultry meat [3]. During the slaughter process, the carcasses can be contaminated by the intestinal contents of infected animals as well as pathogens adhering to the skin [4]. Most cases of salmonellosis are mild and self-limiting in healthy people [5]. However, sometimes it can be life-threatening, particularly in young children, the elderly, and immunocompromised people [6]. The severity of the disease depends on host factors and the serotype of *Salmonella* [7]. The emergence of multi-drug-resistant (MDR) *Salmonella* serotypes is of global public health concern and poses a serious threat to humans through the food chain [3].

There are various control measures, and also safety precautions which have already been taken to reduce the risk of *Salmonella* infection to improve food safety. The European Commission launched an EU-wide *Salmonella* control programme in addition to existing protective measures against *Salmonella* in poultry flocks [8]. The housing and management practices play an important role for animal health and welfare [9,10].

One of the most important management practices affecting animal health and welfare is the litter quality [11,12]. There are many factors affecting the litter quality including ventilation, drinker design, litter material, stocking density, and diet [13,14,15]. The poor litter quality encourages the growth and spread of nonpathogenic and pathogenic microorganisms, which can cause subsequent infection such as *Salmonella* [16]. In addition, litter moisture content has been found to affect microbial survival and activity, including *Salmonella* [17,18]. Many tools have been developed to separate animals from contact with their excreta to reduce the oral infection risk of the animals from contact with contaminated excreta of infected individuals. One of these tools is the use of floor heating to improve the litter quality by reducing its moisture content as stated by Abd El-Wahab et al. [12]. Another approach is the use of slatted flooring. Recent studies evaluated the relationship between slatted flooring and the level of *Escherichia* (*E.*) *coli*, coliform, and antimicrobial resistant *E. coli* [19,20,21].

Feeding strategies could also help to reduce the load of pathogenic bacteria in the bird’s gut. Huang et al. [22] found that feeding a coarse ground meal (average particle size 953 µm) leads to a significantly (*p* < 0.01) faster reduction of *S.* Typhimurium in the contents of the gizzard than when feeding a finely ground meal (average particle size 594 µm). Dietary inclusion of whole wheat or coarsely ground grains demonstrated a decrease in the intestine colonisation with *Salmonella* in broilers [23,24]. Similarly, Ratert et al. [25] showed a significant reduction of *S.* Enteritidis in the caecal content of broilers offered a pelleted diet containing 22% intact whole wheat (geometric mean diameter 422 µm) compared with groups fed finely ground pelleted diets (geometric mean diameter 300 µm). 

Gracia et al. [26] found that mash diets containing whole wheat and oat hull diet significantly reduced the caecal colonization of chickens with *Campylobacter jejuni*. Abadi et al. [27] reported that feeding broilers on pellet coarse diets led to significantly lower caecal *Clostridium* counts. Singh et al. [28] found that the numbers of *Clostridium* as well as *Campylobacter* species decreased with increasing dietary inclusion levels of coarse corn (up to 600 g/kg) in broilers diets.

Nevertheless, despite all the above mentioned strategies to reduce *Salmonella* in poultry, salmonellosis infection is still one of the most common zoonoses in the EU [3]. Furthermore, combatting *Salmonella* contamination in chickens requires detailed knowledge of the major sources of contamination. In order to avoid exposure of the consumer in this regard, minimising the *Salmonella* prevalence at the level of primary production is particularly essential [3,4]. Therefore, it is necessary to gain more precise information concerning its pathogenic properties in relation to the currently most widespread type of housing (floor housing with litter). In particular, the importance of the litter in the spread of a *Salmonella* infection in poultry is of great interest. From another point of view, the dietary measures that promote resistance to the pathogen should be considered. Thus, the present study aimed to investigate potential effects of using floor heating, slatted flooring (decreasing contact between broilers and their excreta), and the different physical forms of the diet (due to different grinding intensity) on the *Salmonella* prevalence and translocation in the liver after experimental infection with *S.* Enteritidis in broiler chickens.

## 2. Materials and Methods

### 2.1. Ethical Statement

All the experiments conducted in this study were approved by the Lower Saxony Ethical Committee for Care and Use of Laboratory Animals (LAVES, Niedersächsisches Landesamt für Verbraucherschutz und Lebensmittelsicherheit; file number 33.12-42502-04-16/2085).

### 2.2. Animals and Housing in Adaptation Phase 

**Figure 1:**
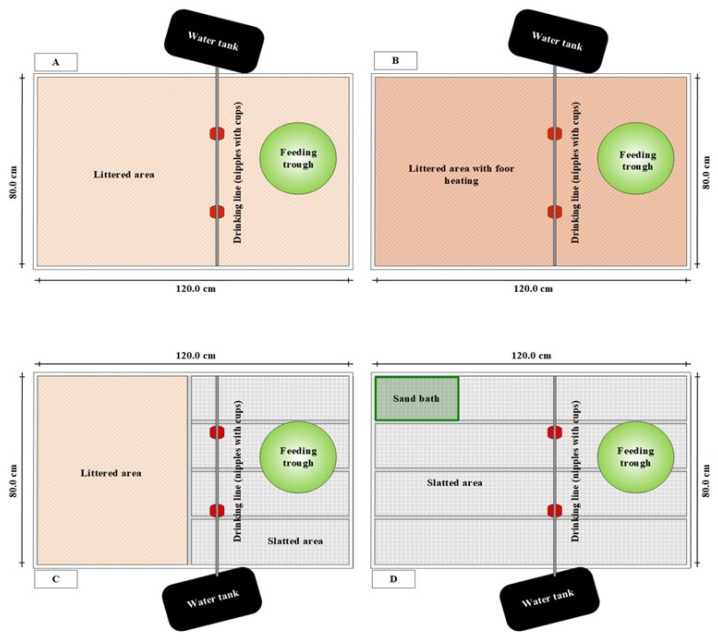
Flooring design of the four different housing systems: (**A**) entire floor pen covered with litter; (**B**) floor pen covered with litter and floor heating; (**C**) partially slatted flooring; (**D**) fully slatted flooring with sand bath.

A total of 480 one-day-old broilers of both sexes (Ross^®®^ 308) were used. The rearing of the birds was divided into an adaptation phase starting from d 1–7 and an experimental period from d 8–37 (Figure 1). During the adaptation phase (d 1–7), the animals were housed in floor pens littered with 1 kg of wood shavings/m^2^ (GOLDSPAN^®®^, Goldspan GmbH & Co. KG, Goldenstedt, Germany). The lighting during the first three days was continuous, this thereafter being decreased to 16 h light and 8 h darkness. The environmental temperature within the stable was adjusted in accordance with the recommendations of the breeding company, Aviagen, Inc. Huntsville, USA [29]. The air and floor temperatures in the box were recorded daily using a temperature logger (EBI 20-TH1, Xylem Analytics Germany Sales GmbH & Co. KG, Weilheim, Germany) and laser thermometer (PCE-777, PCE Deutschland GmbH, Meschede, Germany). Water and the diets were offered *ad libitum* by providing fresh, clean water via nipple drinkers (Big Dutchman International GmbH, Vechta, Germany) and a commercial, pelleted complete feed (GMO STARTER MAXI + W, Best 3 poultry nutrition GmbH, Twistringen, Germany) through an automatic feeder (Crown Suspension Feeder, capacity: 6 kg, Crown Chicken Ltd., Norwich, UK).

### 2.3. Housing and Diet during the Experimental Phase

#### 2.3.1. Housing

At d 8, the animals were moved to the biosafety level 2 infection stable at the Institute for Animal Nutrition of the University of Veterinary Medicine Hannover, Foundation, Germany. Each animal was identified with a wing-tag. In total, eight main groups were formed, with three subgroups of 20 birds each housed in experimental pens with the dimensions 1.20 × 0.80 m. Birds were allocated randomly to 24 groups depending on the flooring (four types) and diets (two types). All boxes were positioned at a distance of approx. 1 m from each other.

The four housing systems differed in their flooring design (Figure 1). In the first housing system (control, L^+^H^-^), the entire floor area was covered with litter (1 kg per m^2^). In the second housing system, (floor heating, L^+^H^+^), the entire floor area was covered with litter and additionally equipped with two heating mats (SAUERLAND GmbH, Paderborn-Elsen, Paderborn, Germany), so that an increase in the litter surface temperature was possible. The litter surface temperature in the group with floor heating varied around 30.0 °C with a difference of 2.70, 3.45, and 3.90 °C for the control, partially and fully slatted flooring groups, respectively. In the third housing system (partially slatted flooring, L^+/−^H^−^), the floor was partially littered (50%) and partially perforated. The floor area was divided in the middle and one half of the box was covered with litter, whereas the other half consisted of plastic slatted flooring in which the diet and water were offered, so that the excreta of the animals in this area fell into a collecting pan below. The slatted flooring consisted of holes (15 × 10 mm) and bridges (plastic covered steel; width 3.5 mm; Big Dutchman International GmbH, Vechta, Germany). In the fourth housing system (fully slatted flooring, L^−^H^−^), the entire floor area consisted of plastic slatted flooring with a sand bath measuring 24 × 37.5 × 6 cm, which was covered with a nest mat (Big Dutchman International GmbH, Vechta, Germany). The sand bath contained about 300 g of sand at the beginning of the experiment at d 8. Thereafter, circa 100 g of sand was added every two days throughout the experiment. 

From d 10 until d 14, all animals received an oral antibiotic treatment with the active ingredient enrofloxacin (Baytril^®®^ 10%, Bayer Vital GmbH, Leverkusen, Germany: 10 mg/kg body weight/day) via drinking water to induce resistance in commensal *E. coli.* [19,20]. 

Before beginning the trial, in order to rule out natural *Salmonella* infection, the animals and the surrounding environment were checked repeatedly up to the experimental infection time to ensure that they were free of *Salmonella* species contamination. For this purpose, all stable areas, boxes, including the litter, sand bath and diets were qualitatively tested before the delivery of one-day-old chicks. In addition, paper inlays on which the first excreta of the chicks were collected and tested. Furthermore, before the start of the experiment at d 8 and during the adaptation period, three samples of manure (litter/excreta mixture) per box were qualitatively analysed for the presence of *Salmonella* at two points of time; first, at d 2 and second at the day before transferring the chicks to the experimental stable (d 7). The same was done with the manure samples at two time-points during the experimental period before inducing infection at d 13 and d 15 (detailed explanations of sampling method are presented in Supplementary Material S1).

#### 2.3.2. Diets

The experimental pelleted diets were offered to broilers ad libitum. The main ingredients in all diets were wheat, soybean meal, and yellow corn, in addition to rapeseed meal during the grower phase; rapeseed meal and triticale were included during the finisher phase. A hammer mill was used to produce the finely ground and coarsely ground diets. The wheat was added in parts intact (unground) to the feed in the coarsely ground group in the following levels: the starter diet contained 3% (first week), the grower diet 5% (second week) and the finisher diet 10% intact wheat. In each fattening phase, the requirements of broiler chickens were achieved by optimising the diet composition. The diets were analysed in accordance with the official methods of the Association of German Agricultural Analytic and Research Institutes [30]. The feed composition and its chemical analysis have been previously reported [31] (Supplementary Material S3).

The wet sieve analysis was performed to measure the particle size distribution for compacted diets in accordance with Wolf et al. [32]. The percentage of particles greater than 2 mm differed markedly between finely and coarsely ground diets in each dietary period. About 17.9%, 11.6%, and 9.66% of particles were >2 mm in finely ground diets for starter, grower, and finisher diets, respectively. In contrast, in coarsely ground diets, the percentage of particle distribution >2 mm was 23.3%, 27.0%, and 29.5% for starter, grower, and finisher diets, respectively. Details regarding the analysis of wet sieve as well as the values of particle distribution for experimental diets have been previously published [31].

### 2.4. Experimental Design, Infection with S. Enteritidis, and Sample Collection

**Figure 2:**
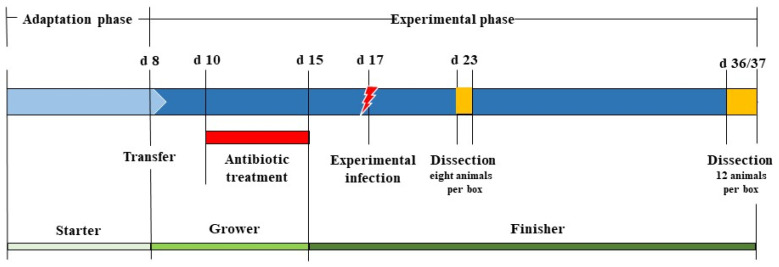
Overview of the trial procedure and sample time plan.

About 48 h after the end of antibiotic treatment (d 17), an experimental infection of two randomly selected birds per box “seeder” with the serovar *S.* Enteritidis (SE147) (at a dose of 8.00 log_10_ colony-forming unit (CFU)/bird) was performed. The strain used was kindly provided and identified by the Poultry Clinic at the University of Veterinary Medicine Hannover, Foundation. To prepare the bacterial suspension, the test strain was streaked on Columbia sheep blood agar (Thermo Scientific™, Thermo Fischer Scientific Oxoid Deutschland GmbH, Wesel, Germany) for 24 h at 37 °C. After incubation to obtain bacterial suspension with a concentration of 8.00 log_10_ CFU/mL, colony material was suspended in phosphate buffer saline ((PBS), Thermo Scientific™, Thermo Fischer Scientific Oxoid Deutschland GmbH, Wesel, Germany) until a McFarland grade of 0.7 was reached (McFarland densitometer DEN-1B, BioSan SIA, Riga, Latvia). To induce experimental infection, the suspension was placed on ice, and 1 mL of the suspension solution was inoculated into the crop of each of the randomly selected birds using a sterile disposable button cannula within 30 min. Simultaneously, the bacterial concentration of the inoculated dose was verified by direct plating of appropriate dilutions of the suspension. Three days post infection, each seeder was qualitatively examined for the presence of *S.* Enteritidis using a cloacal swab in the morning and evening. The spread of the experimental infection to the contact birds was examined at two time-points (d 23 and d 36/37) as part of dissection. The dissection was carried out under all necessary precautionary measures, cleaning and disinfecting the instruments used between animals using (Korsolex^®®^ med AF, BODE Chemie GmbH, Hamburg, Germany). In total, 192 birds (eight per box) at d 23 and 288 birds (six per box and day with a total of 12 birds) at d 36/37 were dissected individually. During dissection, the liver and caecal content were obtained for further qualitative (liver, caecal content) and quantitative (caecal content) detection of *S.* Enteritidis (Figure 2). The qualitative detection of *Salmonella* in the caecal content and liver was performed to determine the prevalence, spread, and liver translocation of *Salmonella* in contact birds. Additionally, the quantitative detection of *Salmonella* in the caecal content, as evidence of caecal colonisation, was performed to assess the actual number of pathogens in the digesta. 

### 2.5. Salmonella Detection, Caecal Colonisation, and Liver Translocation 

#### 2.5.1. Qualitative Detection 

The qualitative examination for *S.* Enteritidis was based on the method DIN EN ISO 6579-1 [30]. Briefly, for wood shavings and feed, samples were incubated in peptone water ((PW), 1:10) at 37 °C for 18 ± 2 h. After incubation, 1 mL of PW was added to 10 mL of tetrathionate brilliant green bile broth ((TBGB), Merck GmbH, Darmstadt, Germany) and simultaneously 0.1 mL was added to 10 mL of Rappaport-Vassiliadis broth ((RV), Thermo Scientific™, Thermo Fischer Scientific Oxoid Deutschland GmbH, Wesel, Germany). Both liquid media were then incubated for 48 h at 42 °C for RV and 37 °C for TBGB. After 24 and 48 h, a loopful (10 µL) of each of the two broth mixtures was streaked onto two selective culture media xylose lysine deoxycholate agar ((XLD), Thermo Scientific™, Thermo Fischer Scientific Oxoid Deutschland GmbH, Wesel, Germany) and Brilliance^®®^ *Salmonella* agar ((BSA), Thermo Scientific ™, Thermo Fischer Scientific Oxoid Deutschland GmbH, Wesel, Germany). The inoculated plates were incubated for 24 ± 3 h at 37 °C. The BSA depended on novobiocin and cefsulodin as a growth inhibitor for other bacteria and the two chromogens caprylate esterase and ß-glucosidase to target specific enzymes produced by *Salmonella* resulted in a purple-coloured colony. Suspected *Salmonella* colonies were purified by subculturing on Columbia agar plates (Thermo Scientific™, Thermo Fischer Scientific Oxoid Deutschland GmbH, Wesel, Germany) and then identified by rapid slide agglutination based on the H and O antigens (anti-*Salmonella* sera, sifin diagnostics GmbH, Berlin) to confirm *S. Enteriditis* as stated below. 

For liver samples, individual liver tissue was immersed in a beaker with ethanol (96%), flamed, weighed, and then placed in a sterile bag (Whirl-Pak^®®^ 710 mL, Nasco International Inc., Fort Atkinson, WI, USA). The organs were minced in a sealed bag using a BagMixer^®®^ 400 (Interscience SARL, Saint-Nom-la-Bretèche, France) for 1 min at speed level 3. Thereafter, the minced liver tissue and other samples (paper inlays with excreta from newly housed chicks, manure and caecal content) were firstly thoroughly mixed and then PW (1:10) was added to each sample. Afterwards, the PW was incubated at 37 °C for 18 ± 2 h. After incubation, three drops of 100 μL each from pre-enriched PW were placed on the top of Modified Semi-solid Rappaport Vassiliadis Medium ((MSRV), Thermo Scientific™, Thermo Fischer Scientific Oxoid Deutschland GmbH, Wesel, Germany) and incubated for a further 48 h at 42 °C. The plates were first examined after 24 h for the formation of a swarm zone. In case of positive plates, a loopful from the outermost edge of the swarming zones was streaked onto the XLD and BS and then incubated at 37 °C for 24 h. Suspected colonies were sub-cultured on Columbia agar (Thermo Scientific™, Thermo Fischer Scientific Oxoid Deutschland GmbH, Wesel, Germany) for purification and serological confirmation.

#### 2.5.2. Quantitative Detection

Quantitative examination of *Salmonella* was performed after dissection for the caecal content of all seeder birds and of all contact birds in both trials, as described by Bast [31]. Briefly, the caecal content was weighed and mixed with PBS (1:10) by vortex (REAX 2000^®®^, Heidolph Instruments GmbH & Co. KG, Schwabach, Germany). In the second step, aliquots (0.1 mL) from sample suspensions ten-fold dilution series were plated in duplicate onto a BS selective agar (Thermo Scientific™, Thermo Fischer Scientific Oxoid Deutschland GmbH, Wesel, Germany). After incubating the media for 24 h at 37 °C, the characteristic *Salmonella* colonies were counted and the results were expressed in log_10_/g caecal content. One suspect colony from each sample was sub-cultured on Columbia agar (Thermo Scientific™, Thermo Fischer Scientific Oxoid Deutschland GmbH, Wesel, Germany) for purification and serological confirmation.

### 2.6. Serological Identification of S. Enteritidis

The serological differentiation of *S.* Enteritidis suspected colonies was carried out by means of rapid slide agglutination in accordance with the manufacturer’s instructions. For this purpose, colony material was picked with a 1 µL inoculation loop and mixed with a drop of isotonic saline solution (NaCl 0.9%, WDT Vision Pharma GmbH &Co. KG, Garbsen, Germany) on a slide which was rotated to rule out autoagglutination. Colony material was then examined in the same way with a drop of the O9 antiserum (sifin diagnostics GmbH). If there was visible agglutination of the antigen with the O9 antiserum, a Kligler agar (Thermo Scientific™, Thermo Fischer Scientific Oxoid Deutschland GmbH, Wesel, Germany) was stabbed with the suspicious colony and incubated at 37 °C for 24 h. If the expected black colouration of the agar occurred as a result of H_2_S formation, the colony was removed from the inclined agar surface for further differentiation and mixed with the antigen sera Hg, Hf, Hm, Hp (sifin diagnostics GmbH), as described above. The antigen formula of the *S.* Enteritidis strain (SE147) used was O:9; H: g, m.

### 2.7. Statistical Evaluation

The statistical evaluation of the results was carried out with SAS Software (version 7.1, SAS Institute Inc., Cary, NC, USA). The qualitative target values concerning *Salmonella* prevalence of contact birds in the caecal content and liver samples were assessed with Pearson’s Chi-square test for homogeneity and for low frequencies with Fisher’s exact test for the factors diet and flooring system. The means of *Salmonella* counts were analysed with respect to the factors diet and flooring system using the Wilcoxon’s two-sample test and Kruskal Wallis test for not normally distributed data. A *p*-value of 0.05 was set as the limit of statistical significance.

## 3. Results

### 3.1. Salmonella Screening before the Start of the Experiment

All swab samples from the stable areas prior to the experiment were examined negative for *Salmonella*. Thus, a natural *Salmonella* infection before d 17 (start of experimental infection) could definitely be excluded by qualitative examination of the paper inlays used in the transport boxes, including the adhering excreta. Furthermore, no *Salmonella* could be detected either in the litter samples at d 2, 7, 13, and 15 or in the feed and sand bath.

### 3.2. Serological Identification of S. Enteritidis 

The rapid slide agglutination test was performed for all the recovered *Salmonella* isolates after experimental infection. This showed that only the applied *S.* Enteritidis strain (O:9; H: g, m) was identified.

### 3.3. Development of Salmonella Infection in the Seeder Birds

In the first three days post-experimental infection, *Salmonella* excretion was confirmed in all seeders at both examined time-points (morning and evening) in the four flooring systems for the birds fed on finely ground diet (100% *Salmonella*-positive cloacal swabs). However, compared to the birds fed coarsely ground diet, lower *Salmonella*-positive swabs were determined (72.3%, 72.3%, 75%, and 83% *Salmonella*-positive cloacal swabs for control, floor heating, partially slatted flooring, and fully slatted flooring groups, respectively) but at least one time-point was positive (Supplementary Material S2 Table S1).

### 3.4. Salmonella Detection in the Seeder Birds at the End of Experiment

The results of qualitative and quantitative detection of *Salmonella* in caecal content and liver of seeders are presented in the Supplementary Material (S2 Table S2). Briefly, qualitative detection of *Salmonella* in caecal content of seeders housed on completely littered floor (control) and fed a finely ground diet displayed 83.3% *Salmonella*-positive caecal content, whereas the seeders from the other three flooring designs exhibited 100% *Salmonella*-positive caecal content. The quantitative level of caecal content contamination ranged from 4.04 to 6.18 log_10_ CFU/g for the birds housed on completely littered or on fully slatted flooring, respectively. Regarding the qualitative detection of *Salmonella* in the liver samples, all flooring designs showed about 66.7% *Salmonella*-positive liver samples. However, the levels of qualitative and quantitative detection of *Salmonella* in the birds fed a coarsely ground diet were lower in comparison to those fed a finely ground diet. The *Salmonella*-positive caecal content varied from 25% in the floor heating to 66.7% in the partially slatted flooring groups. Despite the highest *Salmonella* count observed in caecal content in the birds housed on floor heating (4.88 log_10_ CFU/g), this was still lower than that of birds fed a finely ground diet (5.15 log_10_ CFU/g). The percentage of *Salmonella*-positive liver samples was 100% in the floor heating and partially slatted floor groups in contrast to 50% and 80% for the control and fully slatted flooring groups, respectively.

### 3.5. Salmonella Prevalence and Counts in the Contact Birds

The spread of *Salmonella* infection in the caecal content and the liver of all contact birds was traced through qualitative and quantitative examination at two dissection times. The qualitative detection of *Salmonella* in the caecal content and the liver regarding the flooring design is presented in Table 1. There were no significant differences between the different flooring designs at d 23 for either caecal content or the liver (*p* = 0.5982 and *p* = 0.1927, respectively). Nevertheless, at d 36/37, a significant difference between the different flooring designs did occur for both caecal content (*p* = 0.005) and the liver (*p* = 0.0003). The birds housed on fully slatted flooring displayed the highest prevalence of *Salmonella*-positive samples for caecal content and the liver (88.1% and 91.5%, respectively). In contrast, the lowest *Salmonella* prevalence in caecal content was obtained from birds in the control group (64.4%). Moreover, liver translocations with *Salmonella* in birds reared with floor heating showed the lowest *Salmonella* prevalence (61.7%).

By rearing birds on different flooring systems, no effects were observed in the *Salmonella* count from the caecal content collected at d 23 (*p* = 0.783). On the other hand, there was a significant difference in the *Salmonella* count in the caecal content of birds housed on different flooring designs at d 36/37 (*p* = 0.001), with a mean of 2.21 log_10_ CFU/g *Salmonella* count in the control group compared with 3.76 log_10_ CFU/g in the partially slatted flooring group (Table 2).

The results of the quantitative *Salmonella* detection in the manure or in the sand bath are shown in Table 3. For those animals fed a finely ground diet, the partially slatted flooring design had the highest significant *Salmonella* concentration in the manure (6.13 log_10_ CFU/g) compared to those animals housed with floor heating (4.17 log_10_ CFU/g). In contrast, for broilers consuming a coarsely ground diet, the *Salmonella* count in the manure was similar for the different flooring designs (*p* = 0.239).

The number of *Salmonella*-positive cloacal swab samples from the seeders differed significantly between the birds fed with finely and coarsely ground diets. While *Salmonella* could be detected in 100% of all cloacal swab samples of seeder birds offered finely ground feed, it was found in only 75.7% of all cloacal swab samples from seeders fed coarsely ground feed. The qualitative detection of *Salmonella* from the seeders at the end of the experiment indicates dietary effects regarding susceptibility to *Salmonella* infection. In the finely ground diet group, *Salmonella* could be detected more frequently in the caecal content of the seeders than in the coarsely ground diet group. The quantitative examination of the caecal content of the seeders also showed a higher *Salmonella* count in the caecal content in the broilers fed a finely ground diet than in those animals fed a coarsely ground diet. Regarding the dietary treatment, those broilers fed a finely ground diet showed a higher (*p* < 0.0001) prevalence of *Salmonella*-positive caecal content at d 23 and d 36/37 (97.9% and 93.3%, respectively), compared to those animals fed a coarsely ground diet (30.2% and 58.5% at the respective dissection days; Table 4).

The translocation of *Salmonella* in the liver at d 23 was higher (*p* < 0.0001) in broilers fed a finely ground diet (82.3%) in contrast to those animals supplied with a coarsely ground diet (18.8%; Table 4). This prevalence increased by the end of the experiment (d 36/37) but was still higher (*p* < 0.0001) in birds fed a finely ground diet than those fed a coarsely ground diet (91.7% versus 66.1%; Table 4).

The *Salmonella* counts in the caecal content of broilers fed a coarsely ground diet had a significant difference at both dissection days (*p* < 0.0001; Table 5) compared to those birds consuming a finely ground diet. The highest *Salmonella* count was noted for broilers fed a finely ground diet at d 23 (*p* < 0.0001) compared to those fed a coarsely ground diet (5.07 vs. 0.96 log_10_ CFU/g; Table 5). At d 36/37, the birds fed a finely ground diet had a higher (*p* < 0.0001) *Salmonella* count (3.34 log_10_ CFU/g) in comparison to birds fed a coarsely ground diet (1.94 log_10_ CFU/g).

## 4. Discussion

The present study was conducted to provide more information on the spread and liver translocation of an experimental *Salmonella* infection in broilers exposed to different flooring designs and fed different diets with non-identical particle sizes but of similar ingredients and chemical composition. Successful experimental infection was confirmed for all seeders regardless of the flooring designs and the dietary treatments. In the first three days post infection, the number of *Salmonella*-positive cloacal swabs in all housing systems and for broilers fed a coarsely ground diet was between 26–30 of 36 (72–83%). However, 100% of all cloacal swabs were *Salmonella*-positive in broiler fed a finely ground diet, regardless of the flooring designs. Therefore, it is very likely that the seeders excreted *Salmonella* more frequently in the finely ground diet group than in the coarsely ground diet group. Nevertheless, when comparing the groups, it is impossible to draw conclusions about housing and feeding effects, as *Salmonella* shedding is intermittent and the sensitivity of cloacal swabs for detecting *Salmonella* excretion is insufficiently low [32,33]. Based on the results at the end of the experiment of the qualitative detection of the seeders provided with finely ground (caecal content and liver), no notable differences between the four housing systems were found. However, broilers in the coarsely ground diet group showed differences between flooring designs. In general, based on the test results, it can be assumed that the same conditions prevailed in all groups with regard to the initial experimental infection at d 17, but that there were differences between the trials at the end of the experiment.

### 4.1. Effect of Flooring Designs on Salmonella

Our study’s first hypothesis was that the use of floor heating (30 °C surface temperature) could minimise the survivability and spread of *Salmonella* by drying the litter more effectively. The *Salmonella* count in the manure at the end of the experiment in the floor heating design had almost one log_10_ level less than the control group and around one to two log_10_ levels less than the partially and fully slatted flooring designs. This could be explained on the basis of a surface temperature of 30 °C causing low moisture content of the litter as shown in our recent study [34], where the floor heating design had the highest dry matter content (79%). In turn, the survival and viability of the pathogen in the manure depends on the moisture content of the litter. There are already various indications in the literature that a lower moisture content in the manure can significantly reduce the multiplication and survival of pathogenic bacteria, including *Salmonella* and create a more hygienic environment for poultry production [35,36]. Furthermore, Wilkinson et al. [37] asserted that under experimental conditions, increasing litter temperature leads to a rapid reduction in *E. coli* and *S.* Typhimurium counts in the litter. The results of this previous study [37] support our findings. Therefore, we can conclude that there is a negative association between survival time of *Salmonella* and dry matter content in the litter. Despite floor heating reducing the *Salmonella* count in the manure, there are variations in both qualitative and quantitative determination of *Salmonella* in the caecal content. It is very likely that there is a causal relationship between the number of infected birds and the quantitative shedding of *Salmonella* in the caecal content. It can be assumed that most of the contact birds became infected through the oral ingestion of *Salmonella* contaminated litter material [38]. The amount of *Salmonella* ingested orally determines the proportion of birds whose intestinal tract is colonised with this pathogen [32,39] and also influences the number of *Salmonella* contained in the caecal content [40]. Although the higher surface temperature of the litter led to an increased dry matter content and reduction in the *Salmonella* count, at d 36/37, the *Salmonella* count of the caecal content was higher than that obtained in the control group. This could be due to the *Salmonella* infection from another source than manure. For example, water contaminated with excreta could be a reliable source of infection. An earlier report supported this assumption, stating that *Salmonella* can survive for up to five years in sterile water or a phosphate-buffered solution [41]. In addition, Amaral [42] reviewed that drinking water contaminated with birds’ excreta can transmit diseases to the bird flock. Lamas et al. [43] review the ability of *Salmonella* strains to perform biofilm under different environmental conditions. Therefore, we can conclude that a high consumption of water contaminated with excreta could be an additional source for increasing infection in the floor heating group. The recent study linked with this investigation elucidated that animals housed with floor heating were characterised by a significantly higher water-feed ratio compared to other flooring designs [34].

The second part of the study hypothesis was that the permanent contact of the birds with their own excreta significantly promotes the spread of an experimental *Salmonella* infection through oral ingestion and the recycling of the pathogens. However, there was hardly any excreta accumulating on the slatted flooring and the excreta was collected out of reach of the birds (below the boxes). The results of the *Salmonella* investigations showed a clearly promoting effect of the two housing systems (partially and fully slatted flooring) on the qualitative and quantitative occurrence of *Salmonella* in the caecal content of the contact birds as well as on the translocation into the liver compared to the control and floor heating designs. This may be due to, based on video observations, the pecking behaviour of the broilers when using the slatted flooring, which was focused on the sand bath or ingesting excreta that adhered to the slatted flooring [44]. In addition, slatted flooring covering 50% of the floor area led to a greater spread of the *Salmonella* infection up to d 36/37 compared with the control group. The reason for this unexpected greater spread of the *Salmonella* infection in the contact birds from the partially slatted flooring group could be explained, based on video observations, by the fact that the birds showed a preference for staying and picking in the littered area of the boxes rather than on the slatted flooring [43]. According to the evaluations of the distribution of the birds in the boxes, it can thus be assumed that the contact with the excreta in the partially slatted flooring design was more intensive and of longer duration compared to the fully littered design. The litter quality (assessed on the basis of the dry matter content) was significantly worse in the partially slatted design [34]. Furthermore, since the birds in the partially slatted flooring design preferred to stay in the littered area, more excreta per square metre of littered area was probably deposited in this group than in the control group, where the birds were evenly distributed over the entire floor area. Together with the higher proportion of excreta in the littered area of the partially slatted flooring design, the moisture content of the litter was increased, this probably leading to an increase in the number of *Salmonella*. These findings corresponded with the studies by Dumas et al. [45], who found that the mean abundance of bacteria in wet litter collected from poultry was approximately three times higher than that in dry litter samples. However, neither a partial separation (50% slatted flooring) nor the maximum possible separation of the birds from their excreta (100% slatted flooring) could reduce the spread of the infection when a sand bath was offered. Although both approaches were used to reduce the contact between the birds and their excreta, they led to a greater spread of the infection up to d 36/37. A recent study stated that the litter samples from slatted and litter flooring in broiler houses did not differ in bacterial content, neither in the total bacterial count nor in the *Coliform* or *E. coli* content [21].

### 4.2. Influence of Dietary Treatment on Salmonella

The qualitative detection of *Salmonella* in caecal content and in the liver of the seeders and contact birds at both dissection days differed significantly between the birds fed a finely and coarsely ground diet in spite of a chemically identical composition. This, therefore, indicates the influence of feeding on the animals’ susceptibility to *Salmonella* infection. In addition, the mean count of *Salmonella* in the caecal content also differed significantly between the two dietary treatments (lower *Salmonella* count with coarsely ground diet). Previous data implied that offering a coarsely ground diet may hinder *Salmonella* proliferation [23,24]. Other research discussed the beneficial effect of whole wheat on other bacteria such as *Clostridium perfringens* [46] and *Campylobacter jejuni* [26,47] in broiler flocks. This could be explained on the basis that broilers fed a coarse ground diet showed better gizzard development than those animals fed a finely ground diet [23,34,48]. In addition to the higher relative gizzard mass, various studies also recorded a lower pH level in the gizzard when broilers were fed a coarsely structured diet compared to a finely ground diet [49,50]. Researchers found that coarse feed particles and fiber were retained longer in gizzards [51,52]. This stimulates gizzard activity, and hence increases the production of hydrochloric acid [23,46]. At the same time, the size of particles slows down the passage rate of the feed through the digestive tract, which means that the pathogens are exposed longer to the bactericidal environment [53,54]. This finding is supported by Rodrigues and Choct [55], who elucidated that a developed gizzard can be considered as a barrier, preventing potential pathogenic bacteria from entering the distal digestive tract.

A previous study discussed an additional beneficial effect of offering a coarse compound feed to broilers reflected in the increased synthesis of butyrate in the large intestine [56]. This butyrate can reduce the invasive activity of *Salmonella* [32,57]. There is also evidence that the use of intact cereal grains in compound feed has a positive effect on the microflora in the intestine. Gabriel et al. [58] found that animals that received intact cereal grains excreted more lactobacilli and fewer coliforms. Furthermore, several studies found that coarse particle size corn resulted in a higher caecal count of lactic acid bacteria [27,28]. In this way, the intestinal flora may be stabilised and an infection with *Salmonella* could be prevented due to the competitive exclusion [59]. In contrast, a recent study did not support these findings, suggesting instead that the use of a coarse corn diet alone was insufficient to reduce *Salmonella* prevalence in the caecal content [60]. In addition, Vermeulen et al. [61] concluded that wheat bran with reduced particle size lessens caecal *Salmonella* colonisation when added to poultry feed. Therefore, this enables the conclusion to be drawn that feed structure has a significant influence on the spread of experimental *Salmonella* infection.

## 5. Conclusions

The use of a heating mat (30 °C surface temperature) improved the quality of the bedding by drying the litter material more effectively, which was able to reduce the survivability of *Salmonella* in manure but failed in reducing colonisation of *Salmonella*. Regardless of the flooring system, the feed particle sizes (coarse versus fine grinding) in this study had a significant influence on the reduction of experimental *Salmonella* infection. Therefore, it is critical not only to understand the transmission modes of *Salmonella* in chicken flocks but also to be able to quantitate their relative contribution of each route to contamination during poultry production. Knowing the quantitative contribution of various transmission routes would be very helpful when designing optimal strategies to minimise the spread of *Salmonella*.

## Figures and Tables

**Table 1:** *Salmonella* Enteritidis prevalence at d 23 and 36/37 of contact birds (not experimentally infected) housed in different flooring systems.

Samples	Day	L^+^H^−^	L^+^H^+^	L^+/−^H^−^	L^−^H^−^	*p*-Value
		No. of Positive Samples/No. of Samples (%)				
Caecal content	23	33/48 (68.8) ^a^	29/48 (60.4) ^a^	28/48 (58.3) ^a^	33/48 (68.8) ^a^	0.5982
	36/37	38/59 (64.4) ^c^	41/60 (68.3) ^b,c^	50/60 (83.3) ^a,b^	52/59 (88.1) ^a^	0.0050
Liver	23	28/48 (58.3) ^a^	20/48 (41.7) ^a^	21/48 (43.8) ^a^	28/48 (58.3) ^a^	0.1927
	36/37	45/59 (76.3) ^b,c^	37/60 (61.7) ^c^	52/60 (86.7) ^a,b^	54/59 (91.5) ^a^	0.0003

L^+^H^−^: entire floor pen covered with litter; L^+^H^+^: floor pen covered with litter and with floor heating; L^+/−^H^−^: partially slatted flooring; L^−^H^−^: fully slatted flooring with sand bath. ^a,b,c^ Different subscripts within a row mark significant differences (*p* < 0.05).

**Table 2:** *Salmonella* Enteritidis counts in caecal content at d 23 and 36/37 of contact birds (not experimentally infected, log_10_ CFU/g) housed in different flooring systems.

Day	L^+^H^−^			L^+^H^+^			L^+/−^H^−^			L^−^H^−^			*p*-Value
	Mean ± SD	Max	N	Mean ± SD	Max	N	Mean ± SD	Max	N	Mean ± SD	Max	N	
23	3.44 ^a^ ± 3.17	7.70	48	2.50 ^a^ ± 2.33	8.18	48	2.79 ^a^ ± 2.88	7.41	48	3.04 ^a^ ± 2.98	7.89	48	0.783
36/37	2.21 ^b^ ± 1.75	5.10	59	2.26 ^a,b^ ± 1.76	6.81	60	3.76 ^a^ ± 1.46	7.66	60	2.89 ^a,b^ ± 1.31	5.28	59	0.001

L^+^H^−^: entire floor pen covered with litter; L^+^H^+^: floor pen covered with litter and with floor heating; L^+/−^H^−^: partially slatted flooring; L^−^H^−^: fully slatted flooring with sand bath. ^a,b^ Different subscripts within a row mark significant differences (*p* < 0.05).

**Table 3:** *Salmonella* Enteritidis counts in the manure or the sand bath material (log_10_ CFU/g sample material) in different flooring systems at d 37.

	Finely Ground Diet				Coarsely Ground Diet			
	L^+^H^−^	L^+^H^+^	L^+^/^−^H^−^	L^−^H^−^	L^+^H^−^	L^+^H^+^	L^+^/^−^H^−^	L^−^H^−^ *
Number of samples	3	3	3	3	3	3	3	3
Means (log_10_ CFU/g)	5.30 ^a,b^	4.17 ^b^	6.13 ^a^	5.00 ^a,b^	3.52 ^a^	2.89 ^a^	4.18 ^a^	4.63 ^a^
±SD	1.22	0.15	0.35	0.10	1.41	0.32	1.28	0.54
*p*-value	0.033				0.239			

L^+^H^−^: entire floor pen covered with litter; L^+^H^+^: floor pen covered with litter and with floor heating; L^+/−^H^−^: partially slatted flooring; L^−^H^−^: fully slatted flooring with sand bath. *: in the group L^−^H^−^, the material was obtained from the sand bath. ^a,b^ Different subscripts within a row mark significant differences (*p* < 0.05).

**Table 4:** *Salmonella* Enteritidis prevalence of contact birds (not experimentally infected) of different dietary treatments at d 23 and 36/37.

Sample	Day	Finely Ground Diet			Coarsely Ground Diet			*p*-Value
		No. of Samples	No. of Positive Samples	% of Positive Samples	No. of Samples	No. of Positive Samples	% of Positive Samples	
Caecal contents	23	96	94	97.9 ^a^	96	29	30.2 ^b^	<0.0001
	36/37	120	112	93.3 ^a^	118	69	58.5 ^b^	<0.0001
Liver	23	96	79	82.3 ^a^	96	18	18.8 ^b^	<0.0001
	36/37	120	110	91.7 ^a^	118	78	66.1 ^b^	<0.0001

^a,b^ Different subscripts within a row mark significant differences (*p* < 0.05).

**Table 5:** *Salmonella* Enteritidis counts in caecal content of contact birds (not experimentally infected, log_10_ CFU/g) of different dietary treatments at d 23 and 36/37.

	Finely Ground Diet				Coarsely Ground Diet				*p*-Value
	Mean	SD	Maximum	N	Mean	SD	Maximum	N	
Day 23	5.07 ^a^	2.17	8.18	96	0.96 ^b^	1.69	7.34	96	<0.0001
Day 36/37	3.34 ^a^	1.40	7.66	120	1.94 ^b^	1.73	5.36	118	<0.0001

^a,b^ Different subscripts within a row mark significant differences (*p* < 0.05).

## Data Availability

The data presented in this study are available in this manuscript and the supplementary material.

## Supplementary Materials

The following are available online at https://www.mdpi.com/article/10.3390/microorganisms9040874/s1, Supplementary Material S1: Sampling for qualitative detection of *Salmonella*, Supplementary Material S2 Tables S1 and S2: Salmonella infection determination in the seeder birds three days post infection and at the end of experiment, Supplementary Material S3: Composition of broiler commercial diets during three of the growing phases (g/kg as fed).
