# Supplementary material for: Mitigating the Spread and Translocation of Salmonella Enteritidis in Experimentally Infected Broilers under the Influence of Different Flooring Housing Systems and Feed Particle Sizes
Source: Microorganisms. 2021 Apr 18;9(4):874. doi: 10.3390/microorganisms9040874 (PMC8073070; doi:10.3390/microorganisms9040874)
Supplement: Supplementary file 1 [file microorganisms-09-00874-s001.zip › R1_Microorganism_1187049_Suppl 1.pdf]

Supplementary material S. 1

Sampling for qualitative detection of *Salmonella*

The surface area and box

A smear of the area to be sampled was taken with sterile cotton swabs (Süsse, Hessen, Germany). The swabs then were transferred to a 5 mL peptone water (PW, Thermo Scientific ™, Wesel, Germany) and incubated at 37°C for 24 h.

The litter and feed

Every used package of wood shavings and animal feed was sampled with a sterile sample tube. Then, 25 g from each sample was placed in a sterile bottle with 225 mL of PW and mixed for 30 min at 37°C with a circular shaker (Model 3005, GFL GmbH, Burgwedel). This was followed by incubation at the same temperature for 18 ± 2 h.

Paper inlays

The birds were delivered in perforated plastic boxes with paper inlays "chick diapers" on which the "first excrement" of the chicks were. Each of these baby diapers was shredded with autoclaved scissors, then placed in a sterile Erlenmeyer flask, weighted and mixed 1:10 with PW. The flasks were mixed with a circular shaker and then incubated at 37°C for 18 ± 2 h.

Litter and excrement during adaptation and experimental phase

In order to ensure that no natural *Salmonella* infection had taken place during the adaptation phase (until d 7). Samples were obtained by taking litter material with as large a proportion of fresh excrement as possible from various locations. For each sample, 25 g were placed in a sterile plastic bag (Whirl-Pak®, Nasco, Fort Atkinson) and 225 mL of PW were added. The samples were then mixed for 2 min at level 3 (BagMixer 400®, Interscience, Saint Nom, France) and incubated at 37°C for 18 ± 2 h. During the experimental phase, samples were taken from the d 13 and d 17. In each box, if possible, 25 g of fresh excrement should be taken for further examination. The housing systems with bedding did not always allow a sufficiently large sample to be obtained in a short time. In these cases, parts of the litter were used with the freshest possible excrement additions. The further processing of the samples took place as with the samples from the adaptation phase.

At the end of each cycle (d 37, i.e. after the dissection), the *Salmonella* contents in the litter-excrement mixture or the sand bath was determined. For this purpose, a representative sample of the flat surfaces or the sand bath was obtained from each box and, after homogenisation; 30 g of the sample material were mixed with 270 mL of PW and subsequently examined.
