# Supplementary material for: Mitigating the Spread and Translocation of Salmonella Enteritidis in Experimentally Infected Broilers under the Influence of Different Flooring Housing Systems and Feed Particle Sizes
Source: Microorganisms. 2021 Apr 18;9(4):874. doi: 10.3390/microorganisms9040874 (PMC8073070; doi:10.3390/microorganisms9040874)
Supplement: Supplementary file 1 [file microorganisms-09-00874-s001.zip › R1_Microorganism_1187049_Suppl 2.pdf]

Supplementary material S. 2

Salmonella infection in the seeder birds three days post infection and at the end of experiment

Table 1. *Salmonella* Enteritidis-positive samples of cloacal swabs from the seeders in the first three days of life post infection depending on different flooring systems and dietary treatments

	**Finely ground diet**				**Coarsely ground diet**			
	L^+H^-	L^+H^+	$L^{+/-}H^-$	L^-H^-	L^+H^-	L^+H^+	$L^{+/-}H^-$	L^-H^-
Bird number	6	6	6	6	6	6	6	6
Cloacal Swabs (Npos/Ntot)	36/36	36/36	36/36	36/36	26/36	26/36	27/36	30/36
Percent (%)	100	100	100	100	72.3	72.3	75	83.3

Npos = number of *Salmonella*-positive swab samples; Ntot = total number of swab samples
L^+H^-: entire floor pen covered with litter; L^+H^+: floor pen covered with litter and floor heating; $L^{+/-}H^-$: partially slatted flooring; L^-H^-: fully slatted flooring with sand bath.

Table 2. *Salmonella* Enteritidis-positive samples of the caecal contents and the liver from the seeders on the day 37 depending on different flooring systems and dietary treatments

	**Finely ground diet**				**Coarsely ground diet**			
	L^+H^-	L^+H^+	$L^{+/-}H^-$	L^-H^-	L^+H^-	L^+H^{+1}	$L^{+/-}H^-$	L^-H^{-2}
**Caecal contents**								
Qualitative (Npos/Ntot)	5/6	6/6	6/6	6/6	2/6	1/4	4/6	3/5
Percent (%)	83.3	100.	100	100	33.3	25.0	66.7	60.0
Quantitative (log_{10} CFU/g)	4.04	5.15	4.19	6.18	0.00	4.88	4.34	0.00
**Liver**								
Qualitative (Npos/Ntot)	4/6	4/6	4/6	4/6	3/6	4/4	6/6	4/5
Percent (%)	66.7	66.7	66.7	66.7	50.0	100	100	80.0

N_{pos} = number of *Salmonella*-positive swab samples; N_{tot} = total number of swab samples
L^+H^-: entire floor pen covered with litter; L^+H^+: floor pen covered with litter and floor heating; $L^{+/-}H^-$: partially slatted flooring; L^-H^-: fully slatted flooring with sand bath.

[1] Two seeders died at d 32.

[2] One seeder died at d 32.
