# Supplementary material for: Mitigating the Spread and Translocation of Salmonella Enteritidis in Experimentally Infected Broilers under the Influence of Different Flooring Housing Systems and Feed Particle Sizes
Source: Microorganisms. 2021 Apr 18;9(4):874. doi: 10.3390/microorganisms9040874 (PMC8073070; doi:10.3390/microorganisms9040874)
Supplement: Supplementary file 1 [file microorganisms-09-00874-s001.zip › R1_Microorganism_1187049_Suppl 3.pdf]

Supplementary material S. 3

Table 1. Composition of broiler commercial diets during three the growing phases (g/kg as fed).

**Feedstuff**	**Dietary Phases (g/kg as fed)**		
	**Starter** †	**Grower**	**Finisher**
Wheat *	436	504	461
Soybean meal (42% crude protein)	322	268	251
Corn	145	114	90.0
Rapeseed meal (32% crude protein)	-	19.0	27.0
Triticale	-	-	90.0
Plant oil (soybean)	39.0	48.0	46.0
Animal fat (pig)	9.70	9.50	9.00
Calcium carbonate	17.0	15.0	10.0
Monocalcium phosphate	12.0	8.70	3.30
Sodium bicarbonate	1.80	1.90	1.90
Sodium chloride	1.80	1.40	1.40
Premix **	15.7	10.5	9.40

† The starter diet was identical for all groups and contained 3% whole wheat. * Either coarse (5% or 10%) or fine ground for grower and finisher dietary phases. ** Composition (per kg) for starter diet: methionine hydroxy analogue 3.8 g; vitamin A 11640 IU, vitamin D3 4850 IU, vitamin E 58 mg, zinc 65 mg, iron 52.3 mg, manganese 106.6 mg, copper 14.5 mg, iodine 1.9 mg and selenium 0.29 mg; 6-phytase 485 FTU, 1,4-xylanase 1551 U, narasin 48.5 mg, nicarbacin 48.5 mg. ** Composition (per kg) for grower diet: methionine hydroxy analogue 3.3 g; vitamin A 9500 IU, vitamin D3 4750 IU, vitamin E 33 mg, zinc 47.5 mg, iron 19 mg, manganese 66.5 mg, copper 14.2 mg, iodine 1.9 mg and selenium 0.28 mg, 6-phytase 237 FTU, 1,4 -xylanase 1425 U, narasin 47.5 mg, nicarbacin 47.5 mg. ** Composition (per kg) for finisher diet: methionine hydroxy analogue 2.8 g; vitamin A 12000 IU, vitamin D3 5000 IU, vitamin E 35 mg, zinc 50 mg, iron 20 mg, manganese 70 mg, copper 15 mg, iodine 2 mg and selenium 0.28 mg, 6-phytase 225 FTU, 1,4-xylanase 1350 U, salinomycin-sodium 70 mg.
